# Evaluation of the autogenous bone block transfer for dental implant placement: Symphysal or ramus harvesting?

**DOI:** 10.1186/s12903-016-0161-8

**Published:** 2016-01-26

**Authors:** Selim Ersanli, Volkan Arısan, Elçin Bedeloğlu

**Affiliations:** Department of Oral Impantology, Faculty of Dentistry, Istanbul University, 34093-Capa, Istanbul, Turkey; Department of Oral and Maxillofacial Surgery, Faculty of Dentistry, Istanbul Aydın University, 34088-Florya, Istanbul, Turkey

**Keywords:** Bone defect, Dental implant, Autogenous bone block, Ramus, Symphysis

## Abstract

**Background:**

The absence of sufficient bone volume is the most relevant problem in implant dentistry. Grafting from exogenous sources may provide a limited gain but exhibits poor performance in large bone defects. Autogenous bone block transfer (ABBT) from the mandibular symphysis and ramus has been used with varying rates of success. The aim of this study was to compare the efficacy of symphysal and ramus ABBT for the restoration of lost horizontal alveolar bone volume in the anterior maxilla. Implants placed in the augmented areas were also evaluated.

**Methods:**

The maxillary alveolar bone deficits of 32 patients were treated by similar-sized autogenous bone blocks (7 × 7 × 4 mm) harvested from the symphysis or ramus area. After 4 to 5 months of healing, implants were inserted. At the end of the osseointegration period, the implants were restored by fixed prostheses. Baseline bone thickness was determined by Cone beam computed tomography and was compared to post-op and one-year post-loading bone thickness values where the implants were inserted. Any complications or consequences were noted. The success and survival of the 45 implants were evaluated. The results were analyzed using the Student t-test and Fisher’s exact test (*p* < 0.05).

**Results:**

Post-op complications were frequent in both groups. Baseline bone thickness values were similar at the beginning of the study (*p* = 0.71) and exhibited a significant increase after the ABBT surgery (6.29 (SD 0.86) and 6.01 (SD 0.92) mm in the symphysis and ramus groups, respectively). The amount of bone thickness gain was 4.34 mm (SD: 0.92) and 4.36 mm (SD: 1.01) in the symphysis and ramus groups, respectively. After one year, the mean surface resorption was 0.6 mm (SD: 0.78) and 0.80 mm (SD: 0.56) for the symphysis and ramus groups, respectively (*p* = 0.089). The success and survival rates of the implants were 94.11 and 96.42 %, respectively. No graft failures were observed.

**Conclusions:**

Both symphysal and ramus ABBT procedures were successful for the restoration of a horizontal bone defect in the anterior maxilla. Ramus harvesting may be advisable due to fewer complications. Implants placed in the grafted regions exhibited a high success and survival rate within the one-year follow-up period.

## Background

The lack of sufficient bone volume is one of the major challenges in dentistry. When the placement of dental implants to the anterior maxilla is considered, the restoration of the lost bone volume gets further complicated [1]. Allografts and alloplasts serve a space-maintenance role, whereas fresh frozen transplants confer the risk of disease transmission [2]. Thus, autogenous bone blocks are still considered the gold standard, especially when large volume is required [3]. Iliac or calvarial grafts have been proposed by various studies with varying rates of complications, including infection, mobility impairment and hernia [4, 5]. Furthermore, remarkable surface resorption of the transferred lilac bone graft on the recipient alveolar bone has been reported in many interventions [6, 7]. On the contrary, intra-oral block grafts seemed to be less inclined to long-term surface resorption, conferring improved survival for the osseointegrated implants [3, 8]. However, there remains debate about the donor selection site, and few studies are available regarding the amount of bone gain and long-term surface resorption.

A clinical study was performed to test the following null hypothesis: The amount of bone gain, long-term surface resorption and post-operative complications of block grafts harvested from the mandibular symphysis and ramus regions are not associated with a statistically significant difference. The success and survival of the implants in the restored regions were also evaluated.

## Methods

The study was approved by the ethics committee of Istanbul University (12.01.2013 - R2212). Patients who applied to the university clinic for the treatment of tooth loss between May 2011 and June 2013 were included. A total of 32 patients, consisting of 13 males and 19 females (age range 41–67 years) lacking sufficient alveolar bone volume for the placement of dental implant(s) were included. To eliminate recipient-specific differences, only cases with anterior maxillary deficiencies were included. Initially, all patients were inspected by an intra-oral examination and panoramic x-ray. In the case of visible alveolar bone loss, the patients were further analyzed by a cone beam computed tomography (CBCT) scan to assess the volume of residual bone geometry in cross sections. Patients were informed about other alternative rehabilitation options, and the procedures were initiated upon the written approval of the patient. Only cases with alveolar bone loss were considered, and patients exhibiting significant vertical deformity or a bone defect due to mechanical trauma, pathologic lesions or prior surgical procedures were excluded. To establish an objective comparison, any patients with a local and/or systemic disease that could interfere with the healing process were not included in the study.

### Surgical procedure

Initially, a 0.2 % chlorhexidine mouthwash (Klorherks, Drogsan Pharma, Istanbul, Turkey) was used for intra-oral asepsis. The perioral area was wiped by 10 % Povidone-Iodine (Baticon, Adeka İlaç San. ve Tic. A.Ş, Istanbul Turkey). Infiltration anesthesia was administered. Following a midcrestal incision, a full thickness flap was elevated to reveal two teeth beyond the edentulous area. The flap was extended apically and visually explored.

The decision to use the symphysal or ramus donor area was based on the patient-specific anatomical handicaps, such as the root lengths of the anterior incisor teeth, mouth opening, shallow vestibular sulcus depth and the presence of wisdom teeth.

For the retrieval of the bone-block, infiltration anesthesia was administered to the donor area. In the symphysal area, the horizontal incision was positioned 2 mm apical to the marginal gingiva. Care was given to stay within the attached mucosa for easier suturing and to avoid future loss of the vestibular sulci. Using two vertical incisions extending apically, the donor area was exposed. The superior attachment of the *m. mentalis* was not dissected entirely for better flap repositioning. The dissection was performed in the middle section of the *m. mentalis* attachment, maintaining a 3-mm safety distance from the tooth roots.

In the ramus zone, a midcrestal incision was performed, avoiding the lingual nerve trajectory. The donor area was exposed by extending a full thickness flap in the apical and distal aspect. Care was given to prevent any damage to the *n. lingualis*.

In all patients, a block size of 7 × 7 mm was marked using the piezo electric surgery (Piezon Master, EMS, Basel, Switzerland) and rotational instruments. A block thickness of 4 mm was intended using the depth markers on the periodontal probe (Hufriedy, Chicago, IL, USA) and the piezo-surgery unit’s dedicated surgery tips (EMS, Basel, Switzerland). The block was mobilized manually via surgical chisels. The harvested block was immediately immersed into sterile saline solution to prevent de-hydration. The hemorrhage in the donor bed was controlled by firm gauze pressure, and the flaps were repositioned. In the symphysal region, *the m. mentalis* was repositioned using absorbable 3.0 chromic catgut sutures (Cromik Gut, Alsan, Gaziantep, Turkey). Subsequently, the flap was sutured using interrupted 3.0 silk sutures. A gauze compress was applied to the region for at least 20 min to ensure the establishment of clotting. In the ramus region, the flap was repositioned using 3.0 silk sutures (Doğsan, Trabzon, Türkiye).

**Fig. 1:**
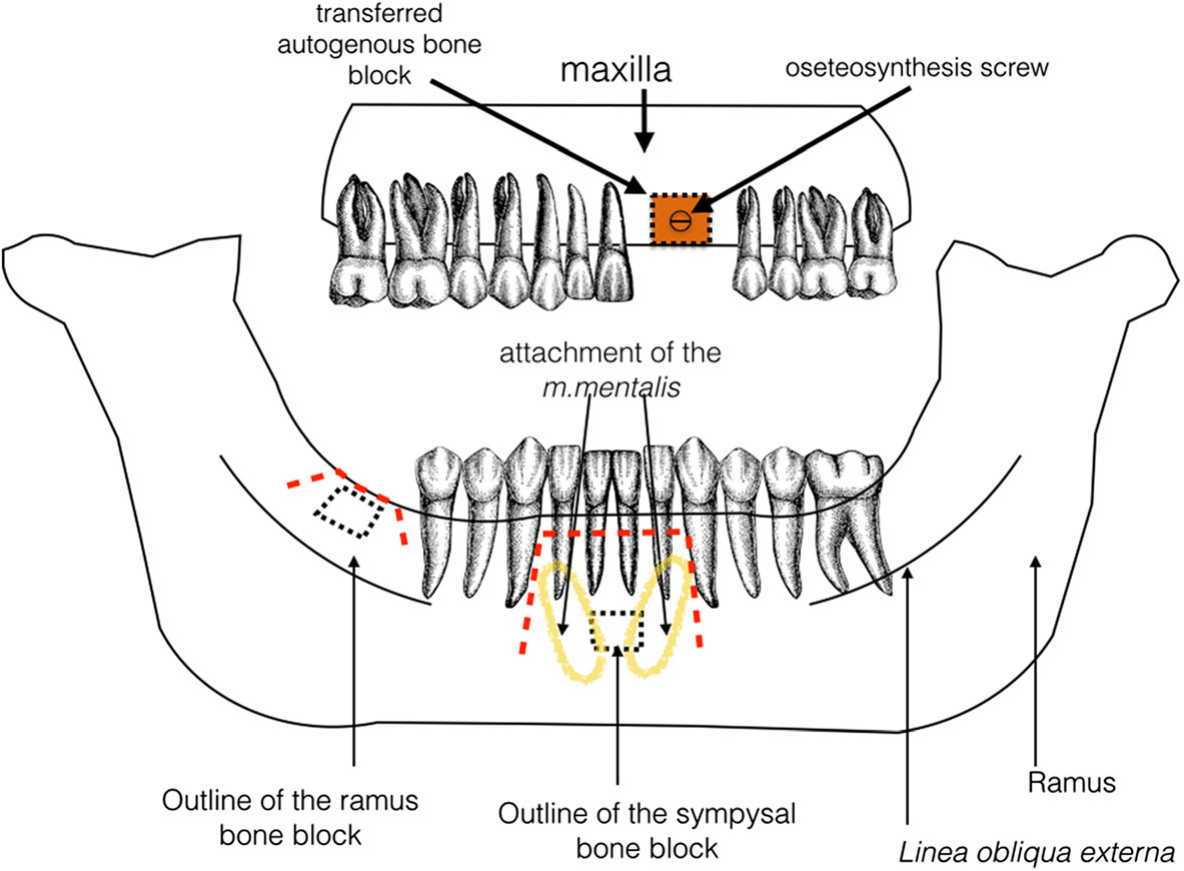
Schematic illustration of the surgical procedures in the study. Lack of horizontal bone volume in the anterior maxilla was treated by bone blocks retrieved from the ramus or symphysis region. Incision lines are represented by red dotted lines

The blocks were slightly trimmed for better adaptation, and one osteosynthesis screw (2 mm diameter and 10 mm width) was used to fix the block bone to the recipient area. A particulate xenograft (Bio Oss, Geistlich Pharma AG, Wolhusen, Switzerland) was used to fill the voids around the block and the recipient bone. A resorbable collagen barrier membrane (Bio-gide, Geistlich Pharma AG, Wolhusen Switzerland) was then laid over the entire grafted area to improve outcomes and reduce the surface resorption of the transferred graft. Any sharp edges or corners were rounded to avoid further soft tissue dehiscence (Fig. 1).

To obtain a tension-free flap closure, horizontally relieving incisions were performed on the apical aspect of the flap until the edges of the wound were brought together. The flap was repositioned by monofilament 3.0 sutures (Vicrly, Ethicon, USA). For the initial control of hemorrhage, sterile saline-soaked gauze was applied over both wound areas. Antibiotics (Amoxicillin & clavulanic acid 1000 mg x2 daily for five days; Klamoks BID, Bilim İlaç, İstanbul, Turkey) and a 0.2 % chlorhexidine mouthwash (Klorheks, Dorgsan Pharma, Istanbul, Turkey) was prescribed to prevent the risk of infection in the post-op period. Patients were instructed to follow meticulous plaque control and a soft diet for one week. The sutures were removed after 10 days, and the block grafts were allowed to heal for four months.

### Implant installation

At the end the healing period, a new CBCT was obtained, and the implant treatment sequence began. All grafts were left to heal for 4 months. At the end of this period, the area was surgically exposed for implant surgery.

**Fig. 2:**
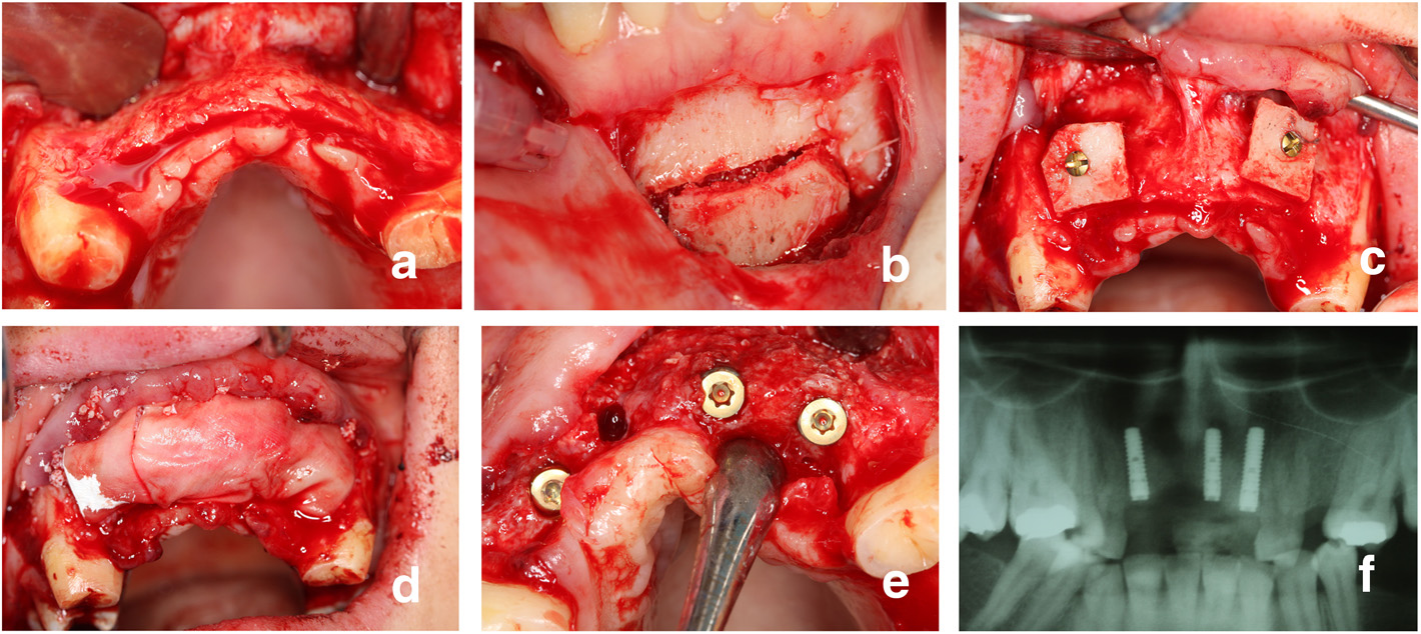
Clinical photographs of a patient in the symphysis group: **a** A full thickness flap was elevated. **b** The block was harvested. **c** The block was secured to the recipient site via an osteosynthesis screw. **d** A resorbable membrane was placed over the graft following the application of a particulate xenograft. **e** The implant was installed after 4 months. **f** Panoramic x-ray after the osseointegration period

The standard surgery protocol was used for the insertion of 45 titanium dental implants (28 and 17 in the symphysis and ramus groups, respectively) of varying lengths and sizes (3.3 to 4.1 diameter and 8 to 13 mm length; Straumann AG, Basel, Switzerland) in the edentulous sites. The implants were allowed to heal for four months, and at the end of this period, the implants were uncovered by punch drills or mini flaps (Fig. 2). The implants were allowed to recover for one month for soft tissue maturation, and impressions were taken after this period. Prosthetic visits were completed within one month, and all implants were restored by metal-ceramic fixed restorations.

### Measurement of the bone thickness changes

CBCT images taken using the same device (Hitachi, CB Mercury, Tokyo, Japan) at the same exposure parameters at three different time intervals (baseline, post-op and 12 moths post-loading) were used for measuring the change in bone thickness in the treatment area via dedicated software (Osirix, Apple, California, USA). The mesio-distal distance from the neighboring teeth (referring the cemento-enamel junction) and the mesio-distal length of the bone block were used to match the consecutive measurements of the area in which the block was placed. Initially, the post-operative image was used to determine the bone-blocks’ exact mesio-distal length and distance to the neighboring teeth. These distances were recorded and used to measure the baseline bone thickness in the baseline CBCT image. The same distance was used to measure the bone-blocks’ surface resorption in the CBCT image taken 12 months after functional loading.

**Fig. 3:**
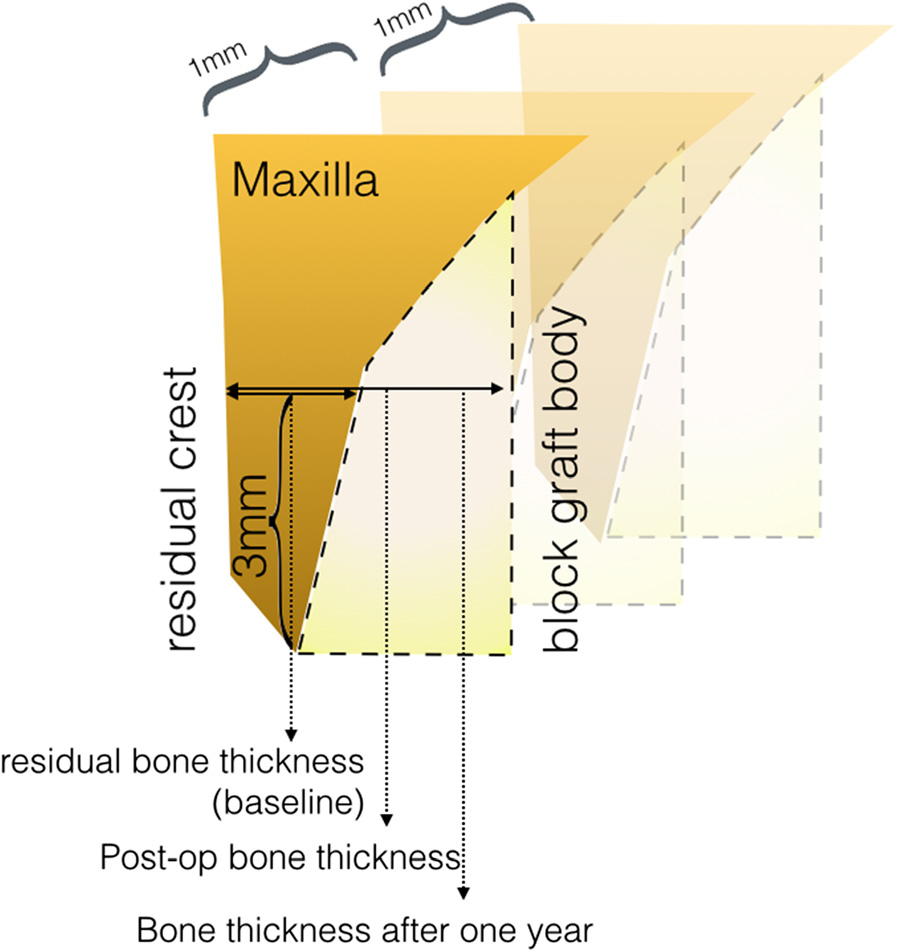
Schematic illustration of the bone thickness measurement

**Fig. 4:**
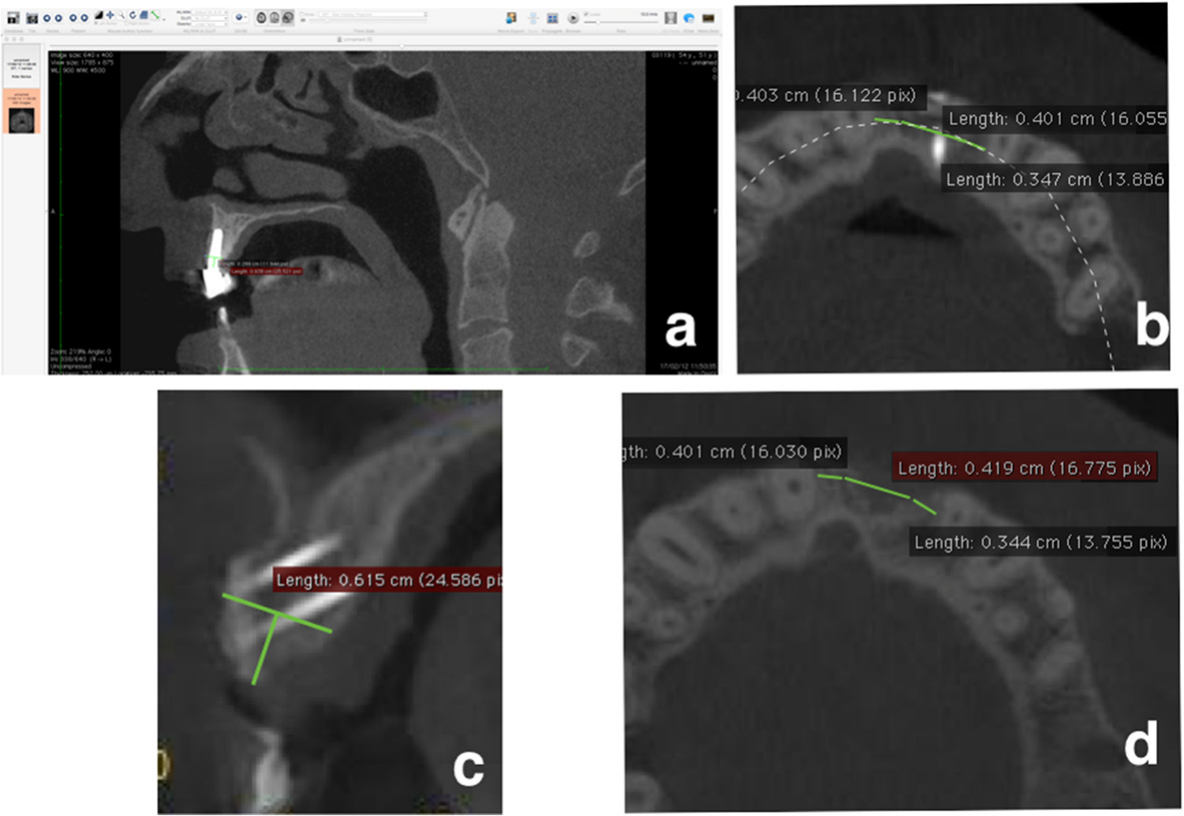
Schematic illustration of the bone thickness measurements over time. **a** Software view of a measurement 12 months after functional loading. **b** To produce a standardized measurement, the disto-mesial distance between the neighboring teeth to the graft body was measured on the post-op CBCT image. This distance was used to determine the thickness of the bone on the first (baseline) and final (12 months after loading) CBCT. **c** All measurements were obtained from a horizontal line drawn from the 3-mm apical point to the tip of the dentulous crest. The distance between the vestibule and the palatinal bone walls was measured in millimeters. This procedure was repeated through the long axis of the graft body for 1-mm slice intervals. **d** Baseline bone thickness was determined from the exact distances measured on the post-op CBCT image

Sagittal and frontal planes are not suitable for mesio-distal measurements over the crestal area. Therefore, a panoramic curve line, which reformats cross-sectional images parallel to and following the curvature of the alveolar process, was adjusted via software. The slice thickness was set to 1 mm. The scout lines were adjusted to visualize the 3-mm apical point of the edentulous ridge. Bucco-lingual bone thickness, referring to the apical 3 mm point of the alveolar crest, was measured on the reformatted sagittal CBCT images. The average of the measurements was recorded as the final bone thickness (Figs. 3 and 4). To assure the accuracy of the measurements, the process was repeated over two separate weeks, and an intra-examiner test (Pearson correlation test) was employed to assure reading reliability. A high reliability was achieved (r = 92.06, *p* = 0.002). Using the above-described methodology, the data retrieved from 32 atrophic alveoli regions treated with 18 symphysis and 14 ramus grafts were included.

The success and survival of the placed implants was evaluated according to the criteria described by Albrektsson and coworkers [9].

### Statistical analysis

Statistical analyses were completed using a software package (GraphPad Prism 5.0, California, USA). The normality of the data sets was confirmed by the D’agtino Pearson Omnibus normality test. The student t test was used for the comparison of bone thickness and peri-implant marginal bone loss measurements. The complication rates were compared using Fisher’s exact test. Reliability of the consecutive measurements was confirmed by the Pearson correlation test. Any *p* value below 0.05 was considered statistically significant.

## Results

All block harvesting interventions were completed uneventfully. When compared with the conventional rotational burs, the use of oscillating piezo-electric surgical instruments was relatively slow but sustained significant control through the osteotomy process. Piezoelectric surgical instrument also allowed trimming and rounding of sharp edges following the fixation of the block on the recipient area.

Post-operative complications were rather high in both groups, although the differences were statistically not significant (83.33 and 78.57 % for the symphysis and ramus groups, respectively; *p* = 0.067) (Table 1). Bleeding was the most frequent post-op complication, followed by the hematoma, flap dehiscence and infection. Furthermore, numbness in the mandibular incisor teeth was reported by 2 patients (13 %) in the symphysis group. A firm gauze compress was recommended to patients complaining of bleeding. For the remaining patients who experienced complications, antibiotics (amoxicillin & clavulanic acid 1000 mg x2 daily) were administered for an additional ten days combined with recommendations for careful plaque control. All complications were resolved except one flap dehiscence, which was treated by a free gingival graft transfer from the palate following the fifth week of surgery. However, one patient in the symphysis group reported continuing numbness of the mandibular incisor teeth after one year. There was no need for block graft removal due to any of mentioned complications.

**Table 1:** Post-operative complications

	Symphysis group (%)	Ramus group (%)
Bleeding	5 (33 %)	4 (36.36 %)
Hematoma	5 (33 %)	4 (36.36 %)
Flap dehiscence	2 (13 %)	2 (18.18 %)
Infection	2 (13 %)	1 (9.09 %)
Numbness	2 (13 %)	0
Total	15 (83.33 %)	11 (78.57 %)

There were no complications in the implant placement surgery. To prevent the risk of graft block detachment from the recipient area, the osteosynthesis screws were not removed. At the end of the healing period, four implants had not osseointegrated, yielding a 91.9 % short-term survival rate. During the course of the study, no further complications or progressive bone loss were noted.

Baseline bone thickness was insufficient for implant placement (1.95 (SD 0.92) and 1.65 (SD 0.79) mm in symphysis and ramus groups, respectively; (*p* = 0.71)) but significantly increased after the block transfer (6.29 (SD 0.86) and 6.01 (SD 0.92) mm in symphysis and ramus groups, respectively). The differences in the bone thickness values between the baseline and post-op period were significant in both groups (t = 12.24 and t = 16.42 for symphysis and ramus groups, respectively, *p* = 0.0001 for both groups).

**Fig. 5:**
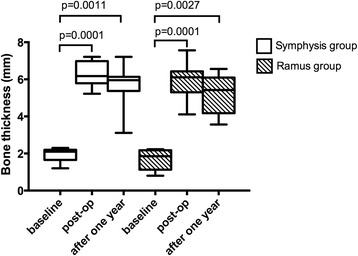
Box-whisker plots of the bone thickness measurements in the symphysis and ramus groups

The amount of surface resorption was 0.6 and 0.86 mm in the symphysis and ramus groups, respectively. When compared with the post-op values, the differences in the surface resorption were not statistically significant (*p* = 0.18 and *p* = 0.29 for the symphysis and ramus groups, respectively). Furthermore, there were no statistically significant differences between the surface resorption values of the symphysis and ramus groups (*p* = 0.089; Table 2 and Fig. 5).

**Table 2:** Mean bone thickness values determined at the measurement intervals

	Symphysis group			Ramus group		
	Baseline	Surgery	After one year	Baseline	Surgery	After one year
Mean (SD)	1952 (.92)	6299 (.86)	5694 (1.77)	1650 (.79)	6011 (.92)	5205 (1.14)

At the end of the healing period, one implant per group had not osseointegrated. At the end of the study period, no implants exhibited further progressive bone loss, yielding 96.42 and 94.11 % survival and success rates for the symphysis and ramus groups, respectively.

## Discussion

The amount of bone gain and subsequent surface resorption by the use of similar-sized ramus or symphysal block grafts were evaluated in this study. The use of tomographic images in small slice intervals through the edentulous crest allowed objective quantification of the bone thickness in all measurement periods. Conventional or optical impression over the soft tissues was not employed because the change in the soft tissue topography may not accurately reflect the underlying bone geometry [10]. Therefore, CBCT scans referring to anatomical landmarks [11] were used for reliable follow-up of the change of bone thickness in the grafted area.

Bone thickness gain through the use of ABBT has been reported to be favorable in many studies. Using a similar methodology Khojasteh and coworkers [12], investigated the bone gain in 102 patients and an average of 4.3 mm (SD 0.93) in the anterior maxilla was reported. The gain was less in the mandible, likely due to decreased vascularization of the cortical structure. The cortical tenting technique has been proposed to enhance bone gain [13]. However, in a clinical split mouth study, block grafts revealed similar bone gain (4.48, SD: 0.51) when compared with the above-mentioned technique [14]. Another study investigated the bone gain and surface resorption of block grafts applied with the same bovine bone material used in this study. The average gain was approximately 5 mm, and at the end of one year, an approximate surface resorption of 17 % was observed [15]. The bone gain observed in this study is consistent with those of other studies and suggests that approximately 4–5 mm of horizontal bone gain can be obtained with the use of the described methodology.

The surface resorption of grafts is an important concern in all autologous grafts. The ilial area has long been used for alveolar bone defects, especially those requiring a large volume. Unfortunately, surface resorption from 5 to 100 % was reported in 42 % of cases. Nevertheless, the iliac grafts incorporate relatively higher trabecular structure and therefore, heal faster than ramus and symphysis grafts. Better healing process also provides resistance to local infection [16]. Calvarial, symphysal and ramus block grafts have been found to be less inclined to long-term surface resorption [3]. However, no objective conclusion could be drawn due to the lack of standardization for objective comparison. Sbordone and coworkers [16] measured the volume changes of 32 autogenous bone blocks and reported an average surface resorption of 35 to 51 % (mean 42 %) at the end of one year. Thus, the resorption rate was higher in the mandible and inversely correlated with the autogenous bone block thickness. In the present investigation, similar-sized bone blocks, which were placed only in the anterior maxillae, allowed an objective comparison. At the end of one year, the surface resorption was not clinically relevant (<1 mm) in either group. This finding is consistent with the findings of Chiapasco et al. [17] and Alerico et al. [18]. Based on the current findings, it may be concluded that the surface resorption of the ramus and symphysal grafts are similar in the anterior maxilla.

As performed in this study, the use of a barrier membrane was proposed to prevent surface resorption by many authors [3, 19, 20]. In addition, Moller and coworkers [6] recommended topical biphosphate application to the collagen membrane for further protection of the long-term graft integrity. The effect of such approaches should be confirmed by further studies to enhance the integrity of intra-oral grafts. It should also be stated the vascularization of the recipient area may have a critical effect on the resorption tendency. Grafts placed in the maxilla yield better results relative to those placed in the mandible and the use of adjunct techniques improving local angiogenesis can be recommended [3, 21].

In addition to the reported satisfactory results, block graft transfers are associated with high complication rates, especially when a vertical component is included. A clinical study of 115 autogenous block grafts revealed only one graft failure. The authors stated that stabilization and intimate contact with the recipient area was crucial to this success. Indeed, no graft failures were observed in the present study, likely due to the use of a vertical component and proper stabilization with an osteosynthesis screw [22]. Nevertheless, other complications that are typical in the post-op period were frequent (approximately 80 % in both groups). Scheerlinck and coworkers [23] harvested block grafts from the iliac, ramus and calvaria and used the grafts for the augmentation of large bone defects in the maxilla and mandible. Complications occurred in more than 64 % of the cases, although the ramus grafted group exhibited the lowest rate of complication. Necrosis of the block graft is the most undesired complication. To decrease the rate of this complication, aggressive decortication of the recipient area is recommended to enhance re-vascularization of the transferred bone graft [22, 24]. In this study, many complications were observed, especially in the post-operative period, and most complications resolved with basic treatment. An annoying neurosensory complication was also observed in one patient, which has also been reported to be specific to symphysal autologous grafts [25]. In contrast, no significant complications were reported in the ramus group; thus, the choice of the ramus may be considered more feasible for ABBT surgery. Based on the current results and those of other reports, it can be concluded that the ABBT procedure is associated with a high rate of post-operative complication. Additional measures and precautions may be advisable for the elimination of the above-mentioned complications after the ABBT surgery. These may include rounding off any sharp corners in the block, a firm fixation to the underlying bone and providing a tension-free flap closure.

Long-term service of osseointegrated implants is necessary following the healing of the ABBT procedures. However, there are few studies reporting the outcomes of osseointegrated implants placed in the autogenous bone block transferred area. In a clinical study involving 192 implants placed into the autogenous bone block, transferred areas yielded a better success rate for one-piece mucosa-level implants (100 %) relative to two-piece bone-level implants. This finding may be particularly relevant in the autogenous bone block grafted areas because the attached mucosa regresses following additional surgical actions for the primary wound closure [26]. The use of implants with a trans-mucosal component yielded better results with respect to mucosa topography, likely as a result of the shifting of the micro-gap to the coronal portion [27]. In general, the survival rates of implants placed into the autogenous bone block transferred area are between 90.01 and 100 % [21], and the success rate is 89.5 to 95.7 % [20]. These figures comply with the results obtained in the present study, and the placement of dental implants into autogenous grafts seems to result in a high survival and success rate. Nevertheless, progressive surface reassertion may eventually risk implant survival in the long term.

## Conclusions

Both symphysal and ramus ABBT procedures can be successfully employed for the restoration of a horizontal bone defect in the anterior maxilla. Both techniques are associated with many complications, and the use of the ramus region may be associated with a decreased incidence of negative outcomes. Clinically irrelevant surface resorption was evident in both groups. A high implant success and survival rate can be expected following the expedition of the described clinical methodology. Further studies should investigate the long-term graft integrity, surface resorption and relevant implant success.

## Abbreviations

ABBT: Autogenous bone block transfer
